# Evaluating the effectiveness of a mobile app-based self-guided psychological interventions to reduce relapse in substance use disorder: protocol for a randomized controlled trial

**DOI:** 10.3389/fpsyt.2024.1335105

**Published:** 2024-05-08

**Authors:** Anna Redeł, Alicja Anna Binkowska, Katarzyna Obarska, Przemysław Marcowski, Karol Szymczak, Karol Lewczuk, Katarzyna Solich, Maria Banaszak, Bohdan Woronowicz, Małgorzata Nowicka, Maciej Skorko, Mateusz Gola, Maksymilian Bielecki

**Affiliations:** ^1^ PredictWatch, Białystok, Poland; ^2^ Nencki Institute of Experimental Biology, Polish Academy of Sciences, Warsaw, Poland; ^3^ Institute of Psychology, Humanitas University, Sosnowiec, Poland; ^4^ Institute of Psychology, Polish Academy of Sciences, Warsaw, Poland; ^5^ Institute of Psychology, The Maria Grzegorzewska University, Warsaw, Poland; ^6^ Institute of Psychology, Cardinal Stefan Wyszynski University in Warsaw, Warsaw, Poland; ^7^ Monar Association, Warsaw, Poland; ^8^ Consulting Center Akmed, Warsaw, Poland; ^9^ Institute of Psychology, SWPS University, Warsaw, Poland

**Keywords:** SUD, addiction, EMA, mHealth, mobile app, cognitive behavioral therapy

## Abstract

**Background:**

Substance Use Disorder (SUD) persists as a significant public health challenge worldwide, with an estimated prevalence of approximately 10-15% across the global populace. This condition is characterized by a notably high risk of lapses and relapses, even subsequent to treatment interventions. Mobile health interventions, owing to their widespread accessibility, emerge as a promising approach to diminish the risk of relapse post-treatment and to broaden the scope of care, especially in regions with a scarcity of trained medical professionals.

**Method:**

This study is designed to assess the effectiveness of mobile interventions in mitigating cravings and preventing lapses among individuals diagnosed with SUD. Employing a two-armed, randomized controlled trial framework, the study will evaluate a self-administered psychological intervention delivered through a mobile application, Nałogometr 2.0. Over a period of three months, participants will engage with intervention modules that primarily incorporate mindfulness techniques and Cognitive Behavioral Therapy (CBT) principles. Ecological Momentary Assessment (EMA) will be utilized to gather longitudinal data on a range of variables that are indicative of craving intensity and the risk of lapse. In addition to this, a monthly-administered battery of questionnaires will be employed to gauge the severity of substance dependence, as well as to measure levels of anxiety, depression, and overall life satisfaction.

**Results:**

Results will be submitted for publication in peer-reviewed journals.

**Clinical trial registration:**

https://clinicaltrials.gov/, identifier [NCT05730504].

## Introduction

Substance Use Disorder (SUD) continues to be a grave concern for public health, affecting an estimated 10-15% of the global population (1). The 5-th edition of the Diagnostic and Statistical Manual of Mental Disorders (DSM-5) lists 11 criteria for an SUD diagnosis, falling into four categories. Criteria 1-4 are related to the impaired control of substance use (consuming the substance in larger quantities and for longer than intended, continuing usage despite the desire to cut down or regulate use, spending a great deal of time on obtaining, using, and recovering from the substance, and experiencing cravings, defined as a persistent desire to use substance). Criteria 5-7 refer to an impairment of social life due to substance use (like inability to fulfill major social and work obligations due to substance use, continuing usage despite it causing significant interpersonal problems, and reduction or discontinuation of social activities because of substance use). Criteria 8 and 9 describe risky use – using the substance in unsafe environments, and persistent usage despite knowing that it may cause physical or psychological problems. Criteria 10 and 11 are physiological: experiencing the buildup of tolerance (requiring increasingly higher doses to achieve the same level of intoxication) and symptoms of withdrawal (adverse effects occuring when substance levels in the body decrease).

Among European countries, Eastern European region has the highest rates of alcohol consumption. Latvia, with 13.19 litres of pure alcohol consumed per capita yearly, is a country with the highest alcohol consumption in the region. In Poland, the yearly consumption is estimated at 11.89 litres of pure alcohol per capita, which is well above recommended use.

Even though in United States less alcohol is consumed (9.97 litres per capita), alcohol dependence rates are similar. It is estimated that 13.9% of US populations struggles with alcohol dependence, which makes US the 5th most affected country (higher rates of alcohol dependence are found in Hungary, Russia, Belarus and Latvia).

In Poland, the most common forms of addiction are alcohol dependence, impacting roughly 12.8% of population (2), and nicotine dependence, affecting around 21% (3). The prevalence of addiction to other psychoactive substances is more difficult to quantify, largely due to legal constraints; however, based on the percentage of individuals seeking treatment, amphetamine (33%) and cannabis (32.8%) are the leading substances of illicit use, followed by opioids (15%) and cocaine (3.1%) (4). The high global prevalence, coupled with the considerable risk of relapse—approximately 40-80% of individuals in recovery return to substance use following treatment completion (5, 6) underscores the particularly chronic and intractable nature of SUD. Morover, a vast majority of those affected, potentially up to 80%, never engage in any form of therapy (7, 8). Contributing factors include a scarcity of medical professionals skilled in treating addiction disorders and patients’ tendency to minimize the severity of their issues. Furthermore, the societal stigma associated with substance use often serves as a barrier, deterring individuals from seeking the help they need (9, 10).

Cognitive Behavioral Therapy (CBT) and mindfulness-based interventions have been established as the most effective methods for treating a range of addictions (11–14). CBT, in particular, demonstrates substantial benefits in addiction treatment as it offers a multifaceted approach by addressing mood disturbances and addiction cravings through the reconstruction of maladaptive beliefs and behaviors, proving its effectiveness in conjunction with pharmacotherapy within clinical settings (15, 16). Additionally, the advent of computer-assisted CBT presents a promising alternative, capitalizing on the ubiquity of computers and internet-enabled mobile devices. Notably, research underscores the utility of a six-module computer-assisted CBT program as a valuable supplement to traditional substance use disorder therapy (17). This innovative solution not only exhibits cost-effectiveness compared to standard CBT but also obviates the necessity for continuous clinician access, marking a significant stride in addiction treatment accessibility.

Mindfulness meditation is recognized for its efficacy in diminishing psychological distress and rumination, as well as reducing symptoms of anxiety and enhancing positive affect (18). Standardized mindfulness interventions have also been shown to significantly alleviate emotional distress and symptoms associated with certain mental disorders (19). Techniques intrinsic to mindfulness, such as focused attention and open monitoring, aim to develop and refine skills in attentional reorientation, metacognition, cognitive reappraisal, and inhibitory control. These competencies are central in effectively managing cravings and maladaptive addictive behaviors (20). Mindfulness-Based Interventions (MBIs) are structured to promote awareness of one’s moment-to-moment experiences, including emotions, thoughts, bodily sensations, and surrounding stimuli. These interventions have demonstrated success in treating SUDs, as they target fundamental mechanisms of addiction by enhancing the recognition and comprehension of triggers, emotions, and thoughts tied to addictive behaviors. MBI programs integrate mindfulness practices to aid in coping with the symptoms of SUD, for instance, by maintaining a mindful presence during experiences of craving in everyday life (21). MBIs have shown greater effectiveness in mitigating withdrawal symptoms and cravings, as well as reducing negative substance use outcomes, in comparison to other psychotherapeutic modalities. However, in terms of substance use frequency and relapse rates, Cognitive Behavioral Therapy (CBT), MBIs, and treatment as usual (*ie, education on substance use, participating in 12-step process-orientated group, or medical management including pharmacotherapy and weekly individual counseling sessions*) did not show significant differences (22).

The emerging sector of mobile health (mHealth) technologies presents a potential strategy to tackle challenges faced by patients with SUD, particularly the heightened risk of relapse following treatment completion. Mobile health encompasses a spectrum of mobile technologies aimed at bolstering health, including mental health (23). Interventions delivered via smartphones are increasingly popular due to their widespread accessibility (24). mHealth interventions have shown promise in augmenting the long-term outcomes of SUD treatments and may play a role in mitigating issues related to social stigma and the insufficient availability of healthcare professionals (25, 26).

Psychological interventions disseminated through mobile technology may offer beneficial therapeutic effects in managing addiction, offering cost-effectiveness and increased accessibility since they do not necessitate continuous contact with a healthcare professional (27, 28). These interventions can be integrated as complementary treatments alongside conventional SUD therapy and pharmacological approaches (17, 29). Additionally, mobile interventions grounded in Cognitive Behavioral Therapy (CBT) can function as supportive measures post-treatment, assisting in the reduction of relapse risk and bolstering therapeutic gains (30).

Mobile technologies facilitate the efficient collection of data via Ecological Momentary Assessment (EMA) (31). EMA entails the real-time and recurrent sampling of participants’ current moods, behaviors, and experiences as they occur in the individual’s natural environment, throughout their daily life (32). While traditional surveys are employed in addiction research, they are often limited to single-time measurements and may not adequately capture the dynamics and fluctuations in behavior (33). EMA not only serves as a research tool for scientists but also supports participants in developing self-monitoring and self- management skills (34), which are recognized as beneficial for behavioral modification in individuals coping with addiction (35–37).

Although interest in mobile health (mHealth) technologies is surging, with over 300,000 applications available in digital marketplaces (38), only a limited number have undergone clinical evaluation before being released for widespread usage (39). Despite many of these applications employing scientific terminology to substantiate their benefits, the evidence supporting them is frequently of poor quality or altogether absent (40). Nonetheless, those apps that have been subject to scientific scrutiny have demonstrated promising outcomes, with positive effects on health-related behaviors (41). The inherent characteristics of mHealth interventions render them an encouraging support mechanism for patients with Substance use disorder (SUD) — given their remote delivery, they can be utilized on demand, providing immediate assistance and support amid episodes of increased craving. Consequently, mHealth solutions are particularly valuable for relapse prevention among patients who have concluded formal treatment. The persistent and considerable risk of relapse following treatment poses a significant challenge in the management of addictive disorders, and mHealth interventions offer a viable approach due to their convenience, ease of use, and broad accessibility. Moreover, mHealth solutions hold the potential to overcome barriers to accessing therapy, such as social stigma, while remaining cost-effective and widely available.

There are some mobile apps dedicated to reducing substance use already available on the market (e.g. *Quitzilla, Drinker’s Helper, Helpic*), but only a few of them have been scientifically tested – examples of such apps include *Drink Less* (42) for alcohol use or *Assess, Plan, Track, Tips (APTT;* 43 *)* and *Norwegian Cannabis Cessation app* (44) for cannabis use. Their features include tracking day-to-day substance usage in a form of sobriety calendars, as well as psychoeducational content on substance dependence and psychological components on recognizing and dealing with triggers and craving.

Given these insights, it becomes imperative to provide patients with addictive disorders access to scientifically validated, evidence-based mobile health (mHealth) solutions. This paper introduces a protocol for a two-arm randomized controlled trial (RCT) designed to evaluate the effectiveness of self-guided, mobile-delivered CBT- and mindfulness-based psychological interventions. The goal is to enhance the post-therapy effects of SUD treatment and aid in the prevention of lapses. These interventions will be administered through the Nałogometr 2.0 app, a science-based mHealth application developed to decrease craving intensity and the risk of lapses in individuals experiencing problematic substance use or those diagnosed with SUD.

The Nałogometr 2.0 app incorporates multiple self-guided psychological interventions predominantly rooted in cognitive behavioral therapy and mindfulness. It offers users the autonomy to interact with any module at their convenience without adhering to a predetermined regimen, thereby affording greater flexibility in tailoring psychological interventions to meet their current needs. Beyond delivering psychological intervention modules, the app is also engineered to foster self-monitoring and self-management by enabling users to self-record their behaviors, and mental and physiological states through Ecological Momentary Assessment (EMA). Additionally, users receive personalized feedback in response to their input.

## Materials and methods

### Aim

The aim of the study is to evaluate the effectiveness of mobile interventions in reducing craving and lapses in patients with substance use disorder.

### Study design

The study was pre-registered within the Open Science Framework (OSF) repository: https://osf.io/z4xqd.

A two-arm participant-blinded randomized control trial will be conducted via a mobile app. The study will compare an intervention experimental condition with a waitlist control condition.

Throughout the duration of the study, participants assigned to the experimental group will have access to self-guided psychological interventions.

Participants will be asked to complete daily EMA questions, as well as questionnaire battery assessments at multiple timepoints: 1) at baseline – in the first week, following onboarding questionnaire; 2) after one month; 3) after two months; 4) after three months.

### Participants

The research will be conducted in collaboration with MONAR and AKMED, Polish organizations responsible for overseeing numerous addiction treatment clinics and centers, as well as other independent institutions. Recruitment of participants will target clinical patients who are undergoing either in-patient or out-patient treatment for Substance Use Disorder (SUD). For the in-patient cohort, the study will include individuals who are in the final stage of their therapy, specifically those expected to conclude their treatment within a maximum of 4 weeks but not fewer than 5 days. Eligible participants must be adults, aged 18 years or older, and must possess fluency in Polish. Moreover, in light of the study’s methodology, enrollment will be limited to users of iOS or Android smartphones.

Participants will be recruited into three groups: 1) Patients with alcohol addiction, 2) Patients with cross-addiction (alcohol and stimulants), 3) Patients with cannabis addiction. We plan on recruiting 150 participants with alcohol addiction, 150 participants with cross-addiction, and ~100 participants with cannabis addiction, due to lower availability of CUD therapeutic programs in Poland – and subsequently, less patients finishing therapy.

Each participant will be assigned to either experimental or control condition upon logging in to the mobile app for the first time.

### Sample size calculation

A simulation-based power analysis was performed to determine the sample size, premised on the use of a linear mixed-effects model with subject-level random intercepts and a small effect size of the intervention. This effect size assumption was informed by literature on the efficacy of mobile interventions in mitigating addictive behaviors (for a review, see 25). The simulation indicated that a minimum of 360 participants would be necessary to achieve an 80% power threshold for detecting a significant group effect. The sampling strategy has been devised to capture maximal demographic variance within the sample, with particular attention to characteristics such as gender, age, geographic location, and socioeconomic status.

### Data collection

During recruitment to the study, participants will be asked to read and sign a consent form. They will also receive necessary study materials: a smartband, along with instructions on how to connect it to their phones, and their individual study code to enter upon logging in to the app.

The research project is based on the principle of complete anonymity, which means that personal data allowing the identification of users is not obtained. For study participants, detailed information about the anonymity of data and how it is processed and stored can be found in the ‘Privacy Policy’ contained in the application and in the ‘Terms and Conditions of Service’. The only demographic data entered into the application are gender, age, and size of place of residence - data we need to create a lapse risk prediction model. The analysis is entirely anonymous - the user will not be compared with the results of any specific user. We plan to conduct comparisons between large groups of users, but these will never be analyses of individual users. Without the user’s explicit consent, we do not share the collected data with entities other than those directly involved in conducting the research and those to which the provision of data will result from a legal obligation. Unless the research participant decides otherwise, the results will be deleted or left in an anonymized form within 30 days from the end of the last phase of the study.

**Figure 1:**
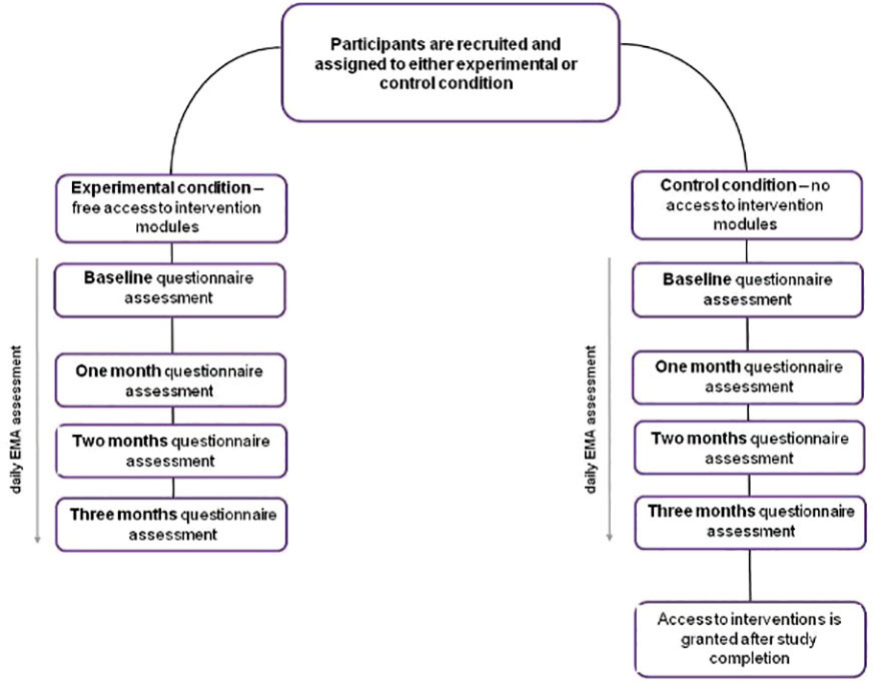
Flowchart of the study design.

Ecological data will be gathered through a mobile application installed on the participants’ smartphones. Upon initial access, participants will encounter a baseline questionnaire designed to collect demographic data and information regarding their patterns of substance use. Throughout the study’s duration, participants will respond to daily EMA prompts and complete a series of questionnaires at specific timepoints, as delineated in **Figure 1**. Throughout the course of the study, the app will be sending daily reminders about EMA prompts, to enhance compliance.

In conjunction with the mobile app data collection, participants will be equipped with smartbands to monitor various physiological parameters, including sleep duration and quality, heart rate, and physical activity levels.

To bolster participant retention rates, an incentive in the form of a contest has been integrated into the study. Participants will be invited to share their experiences with the Nałogometr app, specifically how it aids in modifying their substance use behaviors. The prizes for this contest include a smartwatch and gift cards from the popular polish bookstore, Empik. This motivational component is anticipated to enhance participant engagement and adherence to the study protocol.

### Randomization

Upon their initial login to the mobile application, participants in each group (alcohol, cross-addiction, and cannabis), will be randomly allocated to either the experimental or control condition based on a code they receive during recruitment. Codes corresponding to experimental and control conditions were generated seperately for each group and then put in a randomized sequence. The randomization was done via an online randomization tool (45. [Online] Available from: https://www.sealedenvelope.com/simple-randomiser/v1/lists). Block sizes were determined using the simulation tool (45. [Online] Available from: https://www.sealedenvelope.com/randomisation/simulation/, taking into account planned sample sizes. Participants assigned to the experimental condition will gain immediate access to all psychological interventions following their initial app login. Those in the control condition will have access only to the daily EMA questions and the sobriety calendar, a tool for tracking their substance use. They will be provided access to the complete version of the app, including the psychological interventions, subsequent to the study’s conclusion - which is scheduled for 98 days after the first login.

### Procedure

The study will be conducted with the use of *Nałogometr 2.0*, a mobile app designed to reduce craving and lapse risk in SUD and enhance post-treatment effects (available freely on Google Play and App Store). Prior to the first login, participants will be automatically navigated through all the necessary permissions and consents regarding data collection. Following the app installation, participants will be prompted with an onboarding questionnaire to collect demographic data. They will have a one-week window to complete the initial standardized questionnaire battery assessment. The responses from this initial assessment will serve as the baseline for subsequent evaluations throughout the study. The schedule of these assessments is outlined in **Figure 1**.

During a three-month period, participants will be required to engage in daily EMA assessments and to complete a monthly questionnaire battery. Concurrently, physiological data will be continuously gathered via smartbands.

### Mobile application content

The Nałogometr 2.0 app, available at https://nalogometr.pl/, is designed for individuals seeking to decrease or discontinue their problematic substance use or behavioral habits. The app provides features such as Ecological Momentary Assessment (EMA) and self-guided psychological interventions. For the duration of the study, the accessibility of various modules within the app will be contingent upon the participant’s assigned condition—the control group will not have access to the app’s full capabilities until after the conclusion of the study.

*Dashboard.* The app features a user-friendly dashboard designed for simplicity and ease of navigation, granting users swift access to psychological interventions, EMA modules, and a sobriety calendar, which allows for tracking participants’ substance use.

*Intervention modules.* The application encompasses a suite of self-guided psychological intervention modules. These interventions include a series of audio-guided sessions that focus on gratitude, thoughts management, motivation, relaxation, along with mindfulness sessions aimed at heightening awareness of emotions and bodily signals, and managing stress. These audio sessions are in line with other empirically evaluated self-guided audio exercises (46–48). Furthermore, the intervention module of the app also incorporates CBT-based written exercises that are rooted in thought management and journaling techniques. These are designed to bolster self-confidence and self-efficacy, as well as to deepen the user’s understanding of the interplay between situational triggers, mood, and sobriety. We included longterm intervention modules based on CBT components, e.g., *My beliefs* which is a thought management technique, *Thinking traps*, another thought management and reframing technique, *Planner*, intended to improve goal achievement and self-efficacy, *Mood Journal*, where users are instructed to better understanding of the relationship between situations, thoughts, mood and sobriety, *Dream diary* and *Success diary* intended to enhance self-observation, self-esteem, self-confidence, and awareness of emotions, and *Gratitude Journal* which improves the positive attitude towards yourself, people and world.

Participants assigned to the intervention group will be granted immediate access to all intervention modules upon logging into the app using their unique code. They will have the autonomy to interact with any of the interventions at their discretion without any mandatory commitment. Adherence to a stringent schedule for engaging with the interventions is not a prerequisite for participants in the intervention condition.

### Measures

During the onboarding process—initiated at the first login—participants will be prompted to provide sociodemographic information, including gender, age, place of residence, and details of their addiction profile. They will also respond to questions about their history of substance use, encompassing aspects like the substance involved, duration of use, frequency, treatment history, and periods of abstinence. A series of standardized questionnaires (refer to **Table 1** for an overview) will be employed to gather data on a range of psychological variables. These questionnaire batteries will be administered at one, two, and three months into the study, facilitating the collection of longitudinal data.

**Table 1:** Measures used in questionnaire assessment.

	Group 1 (cross-addiction)		Group 2 (alcohol addiction)		Group 3 (cannabis addiction)	
Measures	Baseline	Monthly assessments	Baseline	Monthly assessments	Baseline	Monthly assessments
*Socio-demographic questions*	**x**		**x**		**x**	
*Onboarding questions*	**x**		**x**		**x**	
Substance use-related measures						
*Dependence severity* (Screener)	**x**	**x**	**x**	**x**	**x**	**x**
*Substance dependence* (SDS)	**x**	**x**	**x**	**x**	**x**	**x**
*Alcohol dependence* (AUDIT)	**x**	**x**	**x**	**x**	**x**	**x**
*Stimulants dependence* (DUDIT)	**x**	**x**				
*Cannabis dependence* (CUDIT-R)					**x**	**x**
Psychological functioning measures						
*Anxiety and depression* (HADS)	**x**	**x**	**x**	**x**	**x**	**x**
*Coping with stress* (mini-COPE)	**x**	**x**	**x**	**x**	**x**	**x**
*Sensation seeking* (BSSS)	**x**	**x**	**x**	**x**	**x**	**x**
*Emotional regulation* (DERS)	**x**	**x**	**x**	**x**	**x**	**x**
*Impulsivity* (SUPPS)	**x**	**x**	**x**	**x**	**x**	**x**
*Life satisfaction* (SWLS)	**x**	**x**	**x**	**x**	**x**	**x**

EMA will be conducted daily to monitor cravings and lapses. The EMA will also record additional variables that are associated with craving intensity, such as current mood, arousal, stress, anxiety, loneliness, fatigue, anger, hunger, and uncertainty.

### Primary outcomes

Primary outcomes of interest will include self-reported number of lapses and addiction craving (intensity of the urge to use a substance at a moment of assessment).

### Secondary outcomes

During the monthly questionnaire assessment, following measures will be applied:

*Substance use.* Self-report psychological measures of substance dependence will be administered across all participant groups. The assessment will include the Severity of Dependence Scale (SDS) (49), which measures dependence on psychoactive substances. Additional questionnaires will be presented depending on the type of addiction. For group A (patients with Alcohol Use Disorder) assessment will include the Alcohol Use Disorders Identification Test (AUDIT) (50). In group B (patients with cross-addiction – alcohol and stimulants) we will administer both AUDIT and the Drug Use Disorders Identification Test (DUDIT) (51). Group C (patients with Cannabis Use Disorder) will complete the Cannabis Use Disorders Identification Test-Revised (CUDIT-R) (52).

*Depression and anxiety.* Symptoms of depression and anxiety among the participants will be quantified using the Hospital Anxiety and Depression Scale (HADS) (53). This instrument is a 14-item questionnaire divided into two subscales: one for anxiety and one for depression, each comprising 7 items. For both subscales, a score ranging from 8 to 10 suggests mild symptoms of depression or anxiety, while scores between 11 and 21 signify the potential presence of a depressive or anxiety disorder.

*Emotion regulation and coping with stress.*Emotion regulation will be evaluated using the Difficulties in Emotion Regulation Scale (DERS) (54), which is a 36-item questionnaire. Participants will rate items on a scale from 1, indicating ‘almost never’, to 5, signifying ‘almost always’. The DERS is organized into six subscales, each designed to assess different facets of emotion regulation difficulties.

For assessing stress management, the Coping Orientation to Problems Experienced (mini-COPE) questionnaire will be used (55). This instrument includes 28 items referring to various coping strategies individuals employ in response to stress.

*Impulsivity and sensation seeking*. Impulsivity will be quantified utilizing the short version of the Impulsive Behavior Scale (SUPPS) (56), which is composed of five subscales. Each subscale contains four items. To assess sensation seeking, the study will employ the 8-item Brief Sensation Seeking Scale (BSSS) (57).

*Life satisfaction*. Life satisfaction will be measured with a 5-item Satisfaction with Life Scale (SWLS) (58).

### Engagement metrics

To assess participant engagement with the Nalogometr application and its components, several metrics will be tracked throughout the study period.

*Average number of days of app usage during the study*: Participants’ engagement with the Nalogometr application was measured by recording the average number of days they accessed the app during the study period. This metric provides an overview of participants’ overall engagement levels throughout the intervention.

*Average number of days of app usage per week*: In addition to the total number of days of app usage, we calculated the average frequency of app usage per week for each participant. This allowed us to assess the consistency of participants’ engagement with the application over time.

*Average number of reminders responded to by the user*: The Nalogometr application includes reminder features aimed at promoting engagement with the intervention components. We tracked the average number of reminders responded to by participants throughout the study, both overall and within specific timeframes.

*Average number of completed interventions:* For participants assigned to the intervention group, the completion of interventions within the Nalogometr application was tracked. We calculated the average number of completed interventions over the study duration and within specific timeframes to evaluate participants’ engagement with the intervention components.”

## Results

### Hypotheses

The primary outcomes of this study are centered on the hypothesis that participants receiving the intervention, as opposed to those in the control condition, will exhibit reduced levels of craving and fewer lapses at the one-month follow-up post-app implementation relative to their baseline levels. It is anticipated that this downward trend in craving and lapse incidents will be sustained throughout subsequent evaluations. Additionally, it is conjectured that a single session of self-guided psychological intervention may yield a notable decrement in both craving intensity and the risk of a lapse occurring. Furthermore, the hypothesis extends to posit that participants in the intervention condition will demonstrate lower levels of substance addiction, reduced symptoms of anxiety and depression, and an elevated sense of life satisfaction when compared to those in the control group, as measured by the relevant standardized questionnaires.

### Data analysis

Factorial design mixed-effects models will be applied to compare questionnaire battery scores between experimental groups and the control across measurements. In addition, we will perform an interrupted time series analysis to estimate the effects of different types of interventions on longitudinal ecological momentary assessment outcomes.

In the statistical analysis, we will incorporate data from participants who have completed a minimum of 21 Ecological Momentary Assessment (EMA) entries, spread over the one-month period dedicated to evaluating the intervention. For the assessment of the intervention’s enduring effects, the analysis will consider participants who have logged at least three EMA entries during the follow-up phase. In addition, within the intervention group, we will include those who have accessed the app and engaged with the self-guided intervention modules on a minimum of four occasions and at least once, respectively; this will represent the minimal therapeutic exposure required for the study. For the analysis of secondary outcomes, we will include participants who have completed the initial baseline assessment as well as at least one subsequent follow-up assessment.

### Data management

Throughout the duration of the research, longitudinal data will be systematically collected via the Nałogometr 2.0 app and securely stored on a protected server. Documentation of this data will comprise codebooks outlining essential information, including data collection protocols, methodological approach, and participant sample characteristics. These codebooks will also detail the types of measures that correspond to each unit of raw data.

The roles of data stewards will be assigned to the Principal Investigators (PIs) and Co-Investigators (CIs), who will oversee the documentation and management of data throughout the processes of collection, analysis, and the eventual dissemination of findings. Team members such as project coordinators, data scientists, and analysts will access the data solely in an anonymized format, adhering to the directives set forth by the PIs and CIs.

After the results have been published, the data will be archived on a server with comparable security measures. Depending on the research phase and the questions being explored, data will be queried and extracted as ASCII files. This anonymized data, including individual participant identifiers, demographic details, and pertinent variable labels and values, will then be made accessible to additional project staff. Subsequently, these team members will undertake any data transformations required to prepare the data for publication-oriented analyses.

Each additional staff member will be obligated to produce documentation describing what data was used and how was it transformed for completing the research task they were involved in. This will include documentation pertaining to the decisions related to any data transformations and coding performed, including variable lists and definitions of the raw data used and how the derived variables were created. Analytical methods and techniques performed for any particular research task will also be documented.

Publications derived from the data collected in this study will strictly utilize anonymized (de-identified) datasets and will focus on presenting results at an aggregate level. Given the anticipated absece of (high) risk to participants, the formation of a data monitoring committee has been deemed unnecessary for this study.

Every additional staff member involved in the research will be required to generate thorough documentation detailing their use and transformation of data in the execution of their assigned research tasks. This documentation will include an explanation of the decisions that guided data transformations and coding, inclusive of comprehensive lists and definitions of the variables derived from raw data, as well as descriptions of the analytical methods and techniques applied in each specific research task. This documentation process ensures transparency and reproducibility of the research findings, and it assists in maintaining the integrity of the data analysis process.

## Discussion

In this protocol, we detail the framework for a two-arm randomized controlled trial aimed at evaluating the efficacy of CBT and mindfulness-based mobile intervention modules administered through the Nałogometr 2.0 app. The objective is to ascertain the impact of these mobile interventions in mitigating cravings and lapses associated with SUD. By examining the utility of the interventions across clinical populations with distinct addiction profiles—alcohol, cannabis, and mixed (alcohol and stimulants) addiction—we aim to gauge the effectiveness of the intervention modules for various manifestations of SUD.

Patients with SUD face a high risk of relapse post-treatment, with estimates suggesting that 40-80% of individuals relapse into addiction following the conclusion of therapy. Mobile psychological intervention modules stand as a potentially powerful means of supporting patient recovery and enhancing the long-term outcomes of treatment. Should the interventions prove effective, they could make a significant contribution to the domain of addiction therapy, representing a valuable asset in the ongoing effort to prevent lapses and relapses.

Furthermore, the study will encompass exploratory analyses aimed at discerning whether the efficacy of the interventions correlates with user engagement levels within the app and subsequent alterations in other psychological domains, such as impulsivity, sensation seeking, stress management, and emotion regulation. These analyses will also consider physical functioning indicators collected through physiological metrics, including heart rate, sleep quality, and activity levels. Delving into this ancillary data will provide deeper insights into the recovery mechanisms from SUD and could inform the enhancement of future health interventions for addictive disorders.

The proposed randomized controlled trial (RCT) will focus on a post-therapy clinical patient population—a demographic that is particularly susceptible to relapse. Conducting the study within the naturalistic settings of the patients ensures the ecological validity of the results and the derived conclusions. Through the collection and analysis of longitudinal data over a span of three months, complemented by regular monthly follow-up assessments, the research aims to elucidate the dynamics of post-therapeutic shifts in cravings and behaviors in patients with SUD.

Regarding the limitations of the study, we anticipate the potential for a high dropout rate, a common occurrence in previous studies (42) and a concern intrinsic to trials with a longitudinal design. Nonetheless, the inclusion of a contest as an engagement strategy is expected to enhance participant retention rates. It’s crucial to note that the opportunity to win prizes is independent of participants’ self-reported levels of craving or instances of lapses, thereby mitigating any potential bias in their responses.

Drop-out rates in mobile application studies on addiction can be high due to various factors, including lack of motivation, technical issues, limited support and guidance, loss of interest, or intervention design. Lack of adequate support, guidance, or encouragement from researchers or healthcare professionals throughout the study can diminish users’ motivation and commitment to using the application. Reducing dropout rates in mobile applications designed for addiction research requires a combination of strategies to increase engagement and enhance the intervention’s effectiveness. Behavioral tracking and personalized feedback can help users stay motivated and focused on their goals. We will use push notifications and reminders to prompt users to engage with the application regularly, complete tasks, or provide updates. We also provide educational materials and psychological intervention modules based on CBT and mindfulness techniques, which could increase the retention rate and provide support in coping with substance use disorder. We informed users at the beginning of the study that the application could not replace psychotherapy.

Drawing from existing literature on mobile interventions for substance use reduction, we are prepared for the possibility of observing only small effect sizes (25). The clinical relevance of a therapeutic intervention is contingent upon externally established standards by researchers and other healthcare professionals. There needs to be more consensus concerning the precise criteria for delineating these efficacy standards. Such criteria may encompass a diminished proportion of treated subjects experiencing adverse outcomes or being susceptible to them, resolution of the targeted issue, or achievement of normative levels of functioning post-intervention. Jacobson et al. (59) delineated clinical significance as a transition towards normal functioning attributable to therapy and outlined methodologies for identifying patients manifesting statistically reliable changes considered clinically significant as per their delineation. Providing a definitive recommendation for a specific effect size that clinicians could universally employ to infer clinical significance poses considerable challenges (60). Despite this, any indication of the mobile interventions’ effectiveness could still be of significant clinical relevance, contributing valuable knowledge to the field of SUD treatment.

## Ethics statement

The study procedures contributing to this work comply with the ethical standards of the Declaration of Helsinki. Ethical approval has been obtained from the Institute of Psychology Polish Academy of Science Ethics Committee (26/XII/2022). The protocol has been registered at a clinical trials database (NCT05730504).

## Author contributions

AR: Writing – original draft, Writing – review & editing. AB: Conceptualization, Methodology, Writing – original draft, Writing – review & editing. KO: Conceptualization, Methodology, Writing – review & editing. PM: Conceptualization, Methodology, Writing – original draft, Writing – review & editing. KSz: Writing – original draft, Writing – review & editing. KL: Writing – original draft, Writing – review & editing. KSo: Conceptualization, Writing – review & editing. MB: Writing – review & editing. BW: Writing – review & editing. MN: Writing – review & editing. MS: Conceptualization, Funding acquisition, Writing – review & editing. MG: Conceptualization, Funding acquisition, Writing – review & editing. MBi: Writing – original draft, Writing – review & editing.
